# Quantitative Research on Digitalized Treatment Options for Older Adults With Mental Illness: Scoping Review

**DOI:** 10.2196/70321

**Published:** 2025-07-07

**Authors:** Jennifer Anne Stanford, Sandra Anna Just

**Affiliations:** 1 Department of Psychiatry and Neurosciences Campus Charité Mitte Charité – Universitätsmedizin Berlin Berlin Germany; 2 Psychologische Hochschule Berlin Berlin Germany; 3 Department of Clinical Medicine Faculty of Health Sciences UiT – The Arctic University of Norway Tromsø Norway

**Keywords:** older people, digital, technology, intervention, psychotherapy, mental illness, dementia, artificial intelligence

## Abstract

**Background:**

Older adults with mental illness face specific physical and psychosocial challenges and inequities, reflected in limited access to advanced technology. This digital divide is alarming as mental health interventions increasingly depend on both patients’ and clinicians’ access to technology. However, digitalized treatments also present opportunities to enhance accessibility, effectiveness, and equity across age groups.

**Objective:**

This scoping review charted the state of quantitative research on digitalized treatment options for older people with mental illness. We focused specifically on how technology is integrated into existing nonpharmacological mental health interventions or leveraged to create new ones. We also summarized the state of the art on the feasibility and effectiveness of these interventions for various mental illnesses.

**Methods:**

This review was conducted in compliance with the PRISMA (Preferred Reporting Items for Systematic Reviews and Meta-Analyses) guidelines for systematic scoping reviews. A PubMed search conducted in April 2024 and updated in April 2025 identified 64 studies (15,644 participants; aged 40-97 years). Included studies were original quantitative studies or reviews of these studies looking into nonpharmacological treatments for older adults with a psychiatric diagnosis using any kind of technology.

**Results:**

The technologies examined ranged from web-based psychotherapy platforms and digital devices for daily challenges to robots for social interaction. Few studies (5/64, 7%) examined the newest advances in digital mental health, such as artificial intelligence or virtual reality. Most studies (37/64, 58%) evaluated dementia-related interventions using small, nonrandomized samples and uncontrolled designs.

**Conclusions:**

The current state of the field, despite the promises of technology to reduce inequities between age groups, still largely excludes older adults from research on technological advances in mental health and their benefits. The field needs to overcome this selective bias and fight the “digital gray divide” in mental health.

## Introduction

### Background

Advances in digital technology in recent decades have created new opportunities for the mental health sector with applications in diagnostics, prevention, and treatment of people with mental illness [1-3]. However, older people with mental illness face a unique set of challenges that may impact the effectiveness of digital interventions. Therefore, this scoping review aimed to investigate (1) which digitalized treatments are available for older adults with mental illness and (2) how effective they are in treating mental illness in this age group.

Digital mental health interventions involve a broad range of technologies, from specifically designed digital interventions such as internet-delivered cognitive behavioral therapy (iCBT) to the use of already available functions such as music apps or SMS text messaging within treatments [4,5]. Previous findings regarding the effectiveness of digital interventions such as smartphone apps, SMS text messaging interventions, and virtual reality (VR) to improve mental health among adults have been encouraging [3,6]. There is ample evidence that iCBT is effective in treating depression and anxiety disorders in the general population [7,8] and it rivals face-to-face therapy in terms of effect sizes [9]. Recent studies seem to indicate a good usability, feasibility, and acceptance of technologies even for patients with serious mental illness [1,10]. For instance, a systematic review on digital technologies in the treatment of psychosis found that most participants used online interventions efficiently and perceived them to be useful [11].

However, technology use is highly correlated with age, a phenomenon that has been described as the “digital gray divide” [12] and that also applies to mental health—older adults with mental illness are less likely to use or even have access to the internet or own a computer or mobile phone [10,13-16]. This inequity in the digital realm likely mirrors inequities in the real world [17-19], with older adults with mental illness facing a specific set of challenges. Barriers to technology use appear to be cognitive deficits and, more specifically, reduced self-efficacy and heightened anxiety with regard to digital technology in older adults [13]. It should be noted that, despite these obstacles, older adults are not necessarily less interested than younger patients in using technology in their treatment and recovery [10,20]. A more general challenge with detrimental effects for the mental health of older adults concerns loneliness—older adults are more likely to live alone, have experienced the loss of loved ones, and have limited mobility and, thus, tend to report higher levels of social isolation and loneliness [21,22]. Both isolation and loneliness can exacerbate existing or contribute to the development of new mental and physical health problems [23-27]. Moreover, lack of social resources may further limit access to mental health services, preventing older adults from receiving timely and appropriate care [28]. However, technology could provide means to overcome some of these social problems and reduce the risk of loneliness (eg, by expanding social networks to the digital world [23,29]). Finally, older adults with mental illness are less likely to seek professional help and are underrepresented in referrals and treatment [30,31]. Commonly identified factors that influence the uptake of psychological treatment among older adults include worries about cost and being prescribed medication, transportation difficulties, and beliefs that illnesses such as anxiety and depression are a normal part of aging [30].

We believe that digital technologies may provide feasible solutions to overcome the identified set of unique challenges of older adults with mental illness. Furthermore, we also need to understand how digital interventions should be designed or adapted to be effective in this age group. Concerning the potential usefulness of digital interventions for older adults, there has been some evidence of technology’s potential to reduce social isolation in the general population, which may, however, vary according to the type of technology and level of use [32]. Moreover, among older adults, use of digital technology appears to be positively associated with health behavior and outcomes [33], potentially including better cognitive functioning [34-38]. In addition, promising results of digital interventions have been found for older adults without mental illness, such as cognitive training using VR, video games, and other software [34,35]. Thus, digital technology presents an exciting opportunity for the treatment of older people with mental illness with their unique challenges, as well as being easily scalable, with the potential for a lower cost per head and reduced burden on in-person mental health services. Concerning older adults’ specific needs when designing digital interventions for this population group, it should be considered that they have a higher rate of multimorbidity and may benefit from a different design and functionality from those for younger users [39]. Significantly, the ability of older adults to manage technology is not dependent just on their own skills but also on factors such as design and clinical diagnosis [40].

### Objectives

Despite the identified need and potential benefit of digital interventions for older people with mental illness, they have previously been underrepresented in research in this field, for example, with only 3% of participants in iCBT studies for depression being aged >65 years [41]. Given the challenges facing older people and their underrepresentation in research on digital technology, this scoping review aimed to provide a comprehensive overview of digital interventions for the treatment of older people with mental illness. This scoping review was guided by the following research questions:

1. What digitalized interventions are available for older adults with mental illness?
2. How feasible and effective are the identified digital interventions for older adults with mental illness according to the current state of research?
3. Which trends and gaps exist in previous research on digital interventions for the treatment of older people with mental illness? Which directions for future research can be identified?

The ultimate goal of this scoping review was to chart the state of the art of digitalized treatment options for older people with mental illness, providing an overview of the existing technologies and summarizing findings on their feasibility and effectiveness for various mental illnesses. This review shall inform clinicians, researchers, and patients alike, enabling decision-making processes for personalized treatments as well as guiding future research in the field.

## Methods

### Overview

This scoping review was conducted in compliance with the PRISMA (Preferred Reporting Items for Systematic Reviews and Meta-Analyses) guidelines for conducting systematic scoping reviews [42]. A preregistration for this review was created in the Open Science Framework [43] before the final screening of studies and data analysis. An overview of the development of the scoping review is detailed in Multimedia Appendix 1.

### Inclusion Criteria

To be included, studies needed to examine (1) the application of digital technology to treat (2) a population aged ≥65 years (3) with a psychiatric diagnosis according to established diagnostic criteria (eg, the *International Classification of Diseases, Tenth Revision*, or the *Diagnostic and Statistical Manual of Mental Disorders, 5th Edition*) and (4) be available in English or German. Importantly, studies were eligible as long as they included participants aged ≥65 years even if the overall sample also comprised younger individuals, meaning that the age range of the participants across the included studies may extend to participants aged <65 years. Qualitative studies, case studies, study protocols, conference proceedings, and studies including no specific information on the sample were excluded from the results. No restrictions were made to publication date.

### Search Strategy

A search of the PubMed database was first conducted on April 30, 2024, and updated on April 28, 2025, by JAS for studies investigating the application of digital technology in the treatment of older adults with mental illness. Only PubMed was chosen due to its comprehensive coverage of health-related literature, for which it is the most widely used and accessible source, and the option to use Medical Subject Heading (MeSH) terms, as well as to limit the scope and ensure the feasibility of this scoping review. The search strategy was built to represent this review’s inclusion criteria in a combination of keywords and MeSH terms:

((digital [Title/Abstract]) OR (technology [Title/Abstract])) AND (((“Psychotherapy” [MeSH]) AND “Aged” [MeSH]) AND (“Mentally Ill Persons” [MeSH] OR “Mental Disorders” [MeSH]))

Using MeSH terms enhances the sensitivity of literature searches by automatically including related concepts and synonyms indexed under the same term. For instance, the MeSH term “psychotherapy” covers a wide array of nonpharmacological interventions ranging from cognitive behavioral therapy (CBT) and psychoanalysis to dance and art therapy.

Moreover, JAS manually examined the bibliographies of the included studies for additional relevant research.

### Study Selection

The results of the search query were uploaded to EndNote (version 20; Clarivate Analytics). JAS and SAJ independently screened the results. In case of disagreement, JAS and SAJ conferred to reach a consensus about study selection.

### Data Extraction and Synthesis

Before conducting the search, outcome variables were defined based on the research questions. In addition to general information on the studies (eg, authors, title, year, and country) and sample characteristics, the type of technology used and measure of effectiveness of technology-based interventions were selected for later data extraction. SAJ conducted the process of data extraction for all tables, whereas JAS extracted the study data for the text of the manuscript. Relevant data from the included studies on study characteristics, type of technology, and effectiveness of the technologies in the treatment of older adults with mental illness were summarized using descriptive statistics and descriptive narration.

After extraction, both authors independently examined the data to identify patterns and recurring use of certain types of technologies. Through an iterative process of discussion, these patterns were developed into preliminary categories, which were then refined into the final set presented in Table 1. The categories were derived inductively from the data, allowing the synthesis to reflect the diversity of the interventions and approaches identified in the literature. Discrepancies in categorization were resolved through consensus, and where needed, an external expert in the field was consulted to ensure the appropriateness and clarity of the final categorization.

**Table 1:** Categories of digitalized treatment options for older adults with mental illness.

Category	Description	Example
iCBT^a^	Online cognitive behavioral therapy programs for depression, anxiety, or insomnia with online lessons, homework, supplementary materials, and interactive features	Managing Your Mood program [44-46]—5 iCBT lessons over 8 wk for people aged ≥60 years with depression
Digital prompts for desired behaviors in patients	Interventions using technology-based media (eg, audio) to prompt desired behaviors in people with dementia	Audio prompts for activities of daily living (eg, washing hands, preparing breakfast, and making coffee) [47-52]
Digital music-based interventions	Interventions for people with dementia involving playing and listening to music via digital devices	Music & Memory [53-55]—personalized music playlists created for people with dementia
Digital media in reminiscence therapy	Reminiscence treatment programs incorporating digital media for people with dementia	Digital life story books containing photos, music, and narration [56]
Digitally simulated family presence	Presence of family members simulated for people with dementia by playing an audio or video recording of the family members	Video in which a family member explains a challenging situation (eg, relative with dementia refusing to take medication) [57]
Digital mental health skill training	Digital training programs teaching older adults with mental illness skills to improve their mental health	DVD training of breathing and relaxation techniques for older adults with anxiety [58,59]
Digital cognitive training	Computer-delivered cognitive training of attention, memory, and visuospatial and verbal skills	Cognitive training games using VR^b^ for people with cognitive disorders [60-62]
Social interaction and activation using robotics	Different types of robots that try to engage people with dementia in social interaction	The most common social robot was the baby seal Paro [63]
Miscellaneous technology	Miscellaneous technology	Wearables, biofeedback, and VR

^a^iCBT: internet-delivered cognitive behavioral therapy.

^b^VR: virtual reality.

## Results

### Search Results

**Figure 1:**
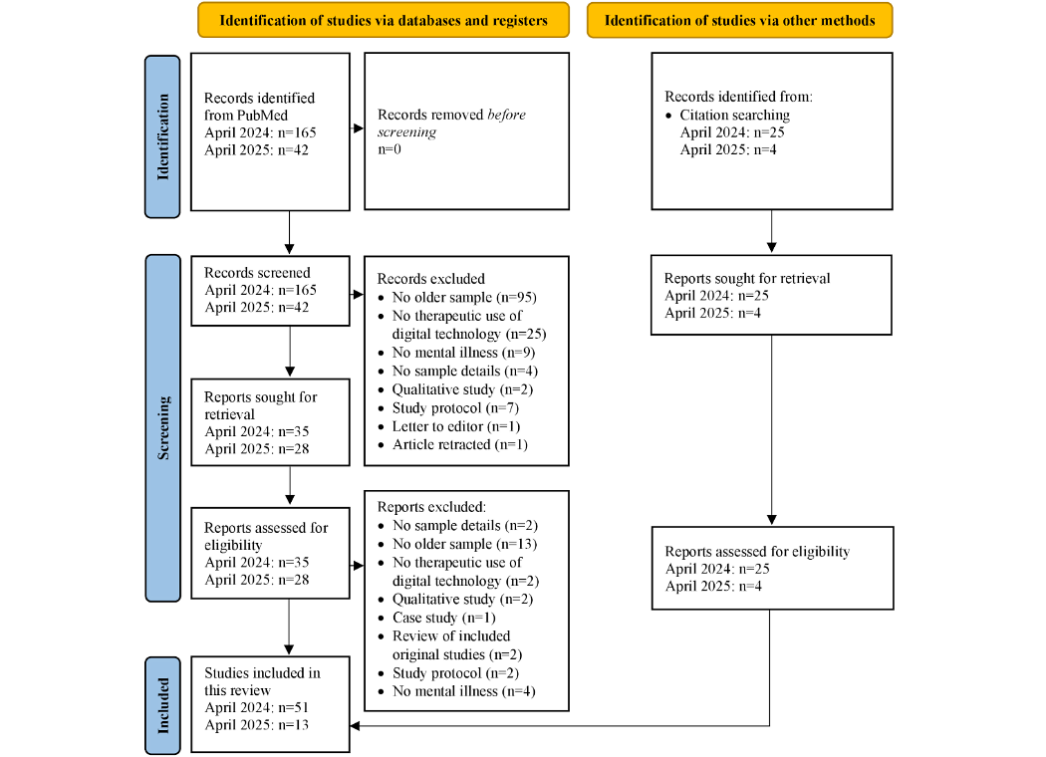
PRISMA flowchart detailing the process of study selection.

**Figure 2:**
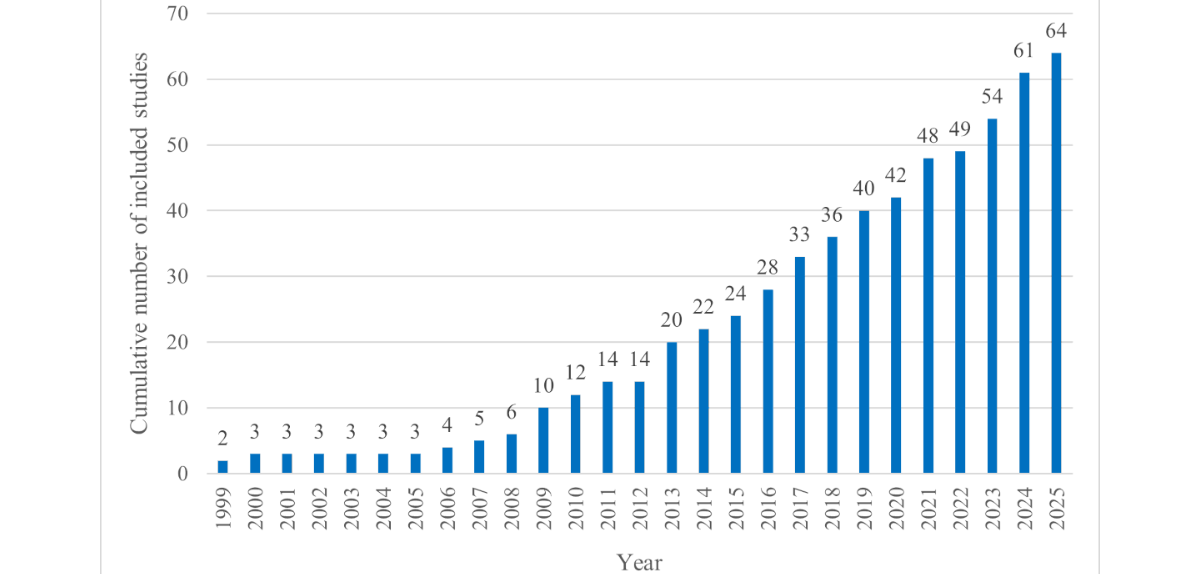
Cumulative number of included studies by year.

The search yielded 207 results (n=165, 79.7% in the initial search and n=42, 20.3% in the updated search). After screening titles and abstracts, the full texts of 63 studies were retrieved for detailed review. Following application of the inclusion and exclusion criteria, of the 63 retrieved studies, 35 (56%) titles remained. The references of the included studies were screened based on the inclusion criteria, leading to the addition of 29 original studies. This brought the total number of studies included in the scoping review to 64. The process of study selection is detailed in a PRISMA flowchart (Figure 1 [64]). An overview of the characteristics of the included studies is provided in Multimedia Appendix 2 [44-53,55-62,65-103] Multimedia Appendix 3 [63,104-109]. Of the 64 studies, more than two-thirds had been published in the last 10 years (n=43, 67% since 2015), with 24 (38%) studies [53,63,65-69,92-107,109] published since 2020. Only 3% (2/64) of the studies had been published before 2000 [70,71] (Figure 2).

Original studies were conducted predominantly in the United States (18/57, 32%), followed by Italy (9/57, 16%) and Australia (8/57, 14%). The total number of participants across all the included original studies was 15,644, with sample sizes ranging between 2 and 12,576. Participants were aged 40 to 97 years, indicating that a lot of studies examining people aged ≥65 years also examined younger populations. Most original studies had small sample sizes, with 89% (51/57) of the studies not exceeding 80 participants. Although randomized controlled trials (RCTs) were common (18/57, 32%), most original studies (31/57, 54%) were conducted without a control condition. We identified nine categories of digitalized treatments in the search results (Table 1): (1) iCBT, (2) digital prompts for desired behaviors in patients, (3) digital music-based interventions, (4) digital media in reminiscence therapy, (5) digitally simulated family presence, (6) digital mental health skill training, (7) digital cognitive training, (8) social interaction and activation using robotics, and (9) miscellaneous technology. The most commonly researched diagnosis in the selected studies was dementia (37/64, 58%), which significantly outweighed the frequency of other investigated diagnoses. These included mild cognitive impairment (MCI; 5/64, 8%), anxiety (11/64, 17%), depression (10/64, 16%), and sleep disorders (4/64, 6%). This notable imbalance in the distribution of diagnoses highlights a clear focus on cognitive disorders, particularly dementia, within the literature. In the following section, the digital technologies available to treat mental illness among older adults are described in detail (first research question). In the subsequent section, the findings concerning the effectiveness of digital interventions are presented (second research question). Table 2 presents the summative results of the included studies by diagnosis.

**Table 2:** Summative results for the included studies by diagnosis (n=57 original studies; n=7 reviews).

Diagnosis	Sample size in original studies	Sample size, mean (SD; range)	Stage of research, n (%)			Most common study design	
			Feasibility	Evaluation	Review	Study design	Studies, n (%)
Dementia (n=37) [47-53,55-57,61-63,65,66,70, 71,77-84,86,87,89,92,96, 100,102-105,107,108]	13,204	412.63 (2184.68; 2-12,576)	9 (24)	23 (62)	5 (14)	Uncontrolled trials	17 (46)
MCI^a^ (n=5) [60,68,91,98,99]	98	19.6 (12.01; 6-39)	3 (60)	2 (40)	0 (0)	Nonrandomized controlled trials	3 (60)
Anxiety (n=11) [45,46,58,59,72,74,76,94,95,101,109]	1707	170.7 (313.53; 10-1095)	1 (9)	9 (82)	1 (9)	RCT^b^	5 (45)
Depression (n=10) [44-46,69,73-76,93,109]	1681	186.78 (325.91; 20-1095)	1 (10)	8 (80)	1 (10)	Uncontrolled trials	4 (40)
Sleep disorders (n=4) [67,88,97,106]	349	116.33 (93.81; 24-245)	0 (0)	3 (75)	1 (25)	RCT, nonrandomized controlled trial, uncontrolled trial, and review	1 (25) each
Other mental illnesses (n=2) [85,90]	30	15 (5; 10-20)	1 (50)	1 (50)	0 (0)	Uncontrolled trial	2 (100)

^a^MCI: mild cognitive impairment.

^b^RCT: randomized controlled trial.

### Which Digitalized Treatment Options Are Being Examined for Older Adults With Mental Illness?

#### iCBT Treatment

The biggest group of included studies (13/57, 23% of the original studies and 2/7, 29% of the meta-analyses) examined the potential of iCBT interventions for older adults with mental illness. Most studies (11/13, 85%) examined iCBT to treat depression and anxiety disorders, reporting medium to large effect sizes for older patients when compared to a waitlist or treatment as usual [44-46,69,72-76,93,101,109]. In addition, some studies (2/13, 15%) found that iCBT interventions to treat insomnia were associated with reductions in both insomnia and depressive symptoms, as well as an improvement in quality of life [67,106]. However, in another RCT on insomnia, offline CBT was superior to iCBT [97]. The quality of the studies on this topic was high, with 38% (5/13) of the original studies being RCTs and 23% (3/13) being nonrandomized controlled intervention studies.

As iCBT builds on the principles of CBT, the interventions included similar common components, such as psychoeducation, cognitive therapy, behavioral activation, problem-solving, and relapse prevention. In addition to the online lessons, the programs were made up of additional features such as homework assignments, reading materials, narration and illustration, and social media features (comments and feeds) [45,46,51,73-75]. Many programs (6/13, 46%) involved therapist support via email or telephone [44-46,72,73,93], but iCBT interventions supported by coaches without specialized mental health training were also effective in treating depression [69]. The programs were developed or adapted for the older target population by including case studies and examples relevant to this age group [44-46,69,72]. Most interventions (8/13, 62%) lasted 8 to 10 weeks [44-46,69,72-75], with 1 outlier lasting 4 weeks [67]. One frequently investigated iCBT intervention was the Managing Your Mood program [44-46] designed for people aged >60 years. The program consists of 5 iCBT lessons for depression over 8 weeks, weekly emails or phone calls from a clinical psychologist, homework between lessons, reminder and notification emails, online resources, and access to comments from previous participants.

Aside from being effective, findings indicated that iCBT could be translated to real-world settings [76] and be cost-effective [46]. The interventions were well accepted by participants [44-46,69]. Older participants were as likely to enroll in an online intervention as younger people, and their adherence was higher than that of younger people [76]. Finally, allocating treatment after identifying patients who might benefit more from online or offline interventions appeared to further improve the effectiveness of iCBT interventions [97].

#### Digital Prompts for Desired Behaviors in Patients

The second largest group of studies (13/64, 20%) looked into interventions using technology-based media to prompt desired behaviors in patients. The studies’ research focus was dementia and cognition. Several studies (5/13, 39%) found that audio prompting led to a significant improvement in the ability of people with dementia to complete activities of daily living such as washing hands, preparing breakfast, or making coffee [47-52]. The technology used was simple—a photocell and light-reflecting paper to create a light beam, which was broken when a participant reached for an object, as well as a Walkman to play audio prompts [48,51,77]. Encouraging initial results were maintained over a 6-month period in a small sample [48]. There was also preliminary evidence of the potential of this technology to improve the mood of participants, with 15% (2/13) of the studies finding significant increases in behavioral indicators of happiness after completing an activity as compared to other activities [48,50]. Another 15% (2/13) of the studies found that auditory cues could lead to an improvement in participants’ ability to travel from one room to another by themselves [49,78]. In total, 8% (1/13) of the studies found that video prompts were more effective than audio prompts [47].

Another study used assistive technology with artificial intelligence (AI) to deliver art therapy via a computer-based system to help engage people with dementia in the activity and provide them with visual or audio prompts if they became distracted. Despite positive ratings of the system among participants and therapists, it was felt that the prompts were ineffective in recapturing the attention of participants [79].

Another study examined how people with dementia could be enabled to grow and take care of plants in their homes using a smart grower, app, and social media support [96]. The intervention improved the well-being of participants and their caregivers; however, technical difficulties were identified as an obstacle, and the intervention was not compared to offline interventions.

Finally, 15% (2/13) of the studies examined how openly available, nonspecialized iPad apps could be used to prompt group activities for people living with dementia. The iPad apps (defined as “mentally stimulating” [66] or not further specified [80]) were found to offer an acceptable alternative to or extension of standard group activities such as cooking or crafts [66,80].

#### Digital Music-Based Interventions

Digitalized music-based interventions were examined in 9% (5/57) of the original studies and 14% (1/7) of the meta-analyses. These interventions were based on the assumption that playing and listening to music via digital devices would have a positive effect on people with dementia. A total of 40% (2/5) of the studies found that listening to music was associated with decreased agitation and apathy among people with dementia [65,81]. One study looked at 2 devices: a music device that allowed older people with Alzheimer dementia in a day care center to select nostalgic songs and a video device for selecting video clips, both seemingly promoting engagement and positive affect and reducing fidgeting [82]. Guided and individualized interventions were accepted better by participants than nonguided interventions [65]. The widely adopted Music & Memory intervention [54], in which personalized music playlists are created for people with dementia, showed mixed results—the findings of a large (N=12,576) retrospective analysis, comparing nursing homes that adopted Music & Memory with those that did not, that these playlists were associated with reduced medication rates and problematic behaviors [55], could not be replicated in a recent RCT [53]. The meta-analysis could not find a beneficial effect of music-based interventions on the mood and cognition of people with dementia [107].

#### Digital Media in Reminiscence Therapy

We defined this category as any reminiscence intervention incorporating digital media into a common and well-researched treatment approach for people with dementia that aims to activate patients’ resources, evoke memories, and improve well-being [110]. In total, 43% (3/7) of the works found on the topic were original studies [56,83,84], 43% (3/7) were systematic reviews [104,105,108], and 14% (1/7) were scoping reviews [63]. All studies (7/7, 100%) examined the potential of multimedia interventions to alleviate psychological and behavioral symptoms associated with dementia. Exploiting digital technology for reminiscence therapy by including personalized digital photos, videos, and music as well as digital life story books seemed to have advantages tailored to the specific challenges of older adults with dementia (eg, the content was more accessible to people with motor and sensory deficits or promoted more personalized engagement and a greater sense of autonomy than traditional reminiscence materials [56,83,108]). Overall, findings concerning improvement in quantitative health outcomes such as mood and cognition through multimedia interventions were scarce and inconclusive [63,108]. The use of digital life story books was associated with improved mood and cognition in a small study, but no tests of significance were conducted [56]. A systematic review indicated that the personalized nature of the content was central to its success [105]. In a comparison of a personalized intervention with a generic news program, people with dementia demonstrated significantly higher levels of concentration and lower levels of distraction when watching a personalized video [83]. However, nonpersonalized content also showed potential as a tool to promote communication between people with dementia and caregivers and to enable people with dementia to play a more active role in determining the focus of a conversation [84].

#### Digitally Simulated Family Presence

Another use of technology and media in treatments for people with dementia was simulated presence (3/64, 5% of the studies), either by playing an audio excerpt of a family member made to resemble a telephone call or a video excerpt of a family member on a tablet. In people with dementia, simulated presence was associated with reduced verbal and physical agitation [71,81], and short videos recorded by family members were superior to treatment as usual and placebo conditions in bringing about changes in agitated and withdrawn behaviors [71]. Videos of family members explaining preidentified challenging situations (eg, their relative with dementia refusing to take medication) appeared to help care professionals in resolving these situations [57].

#### Digital Mental Health Skill Training

Several studies (5/64, 8%) evaluated digital programs designed to teach older adults with mental illness skills that can improve their mental health. A feasibility study demonstrated high usability of an app designed to train older adults with serious mental illness in self-management methods [85]. Another study investigated whether older adults with MCI or subjective cognitive decline would use a tablet-based app for practicing meditation at home between in-person sessions of a mindfulness-based stress reduction course. Overall, low adherence rates did not differ between participants with and without tablets, but participants using tablets appeared to accept the technology and did not prefer switching to CDs [68]. The last examined intervention was developed to decrease symptoms of anxiety in older adults. The video-delivered intervention aimed to teach diaphragmatic breathing and progressive muscle relaxation in weekly sessions via DVD, making it accessible to people without an internet connection [59]. It was developed by Gould et al [58], who investigated it in 4 studies, reporting significant reductions in anxiety, depressive, and somatic symptoms but no significant effect at follow-up [94] or in comparison to psychoeducation [95]. While the intervention was considered feasible, the requirement for self-managed home practice was identified as a barrier to its effectiveness [95].

#### Digital Cognitive Training

Of the 64 studies, we found 11 (17%) on the potential of technology-based cognitive training for people living with a diagnosis of dementia or MCI. Of these 11 studies, 4 (40%) looked at computer-delivered trainings that ranged in length from 10 sessions in total [70] to 3 sessions per week over 24 weeks [86] and trained functions such as attention, memory, and visuospatial and verbal skills [70,86,87], with some studies (2/11, 18%) examining online adaptions of cognitive stimulation therapy [92,102]. The interventions were designed with multiple levels of increasing difficulty [86,87]. One study identified a significant improvement in specific language-related measures following the training [87], whereas another study indicated a tendency toward improvement in this area that was not significant [86]. The findings regarding an association with memory were mixed, with one study finding a positive association between the training and visual and topographical memory [70] and another study, which specifically measured memory, not yielding significant results [86]. There are initial indications that computer-based cognitive training may be associated with improved affect, with one study finding a significant effect on symptoms of depression [87].

A total of 27% (3/11) of the studies looked at the application of VR to train cognitive functions in people with dementia or MCI [60-62]. VR has potential as a training environment with ecological validity, which theoretically makes it possible to train skills that are applicable in the real world [61]. The applied VR technology varied among the studies. One study let patients with dementia take part in virtual cognitive training games via a computer screen and controllers, which were able to provide sensory feedback [62]. Only 1 cognitive training, the GRADYS game, used a head-mounted display for their VR application [61]. The different VR environments have been found to have good usability and acceptability among older patients with cognitive impairment [60-62]. The VR-based cognitive trainings aimed to train functions such as attention, memory, and visuospatial skills and were associated with significant improvements in cognitive skills among healthy people [61]. However, 67% (2/3) of these interventions did not establish a statistically significant change in this area among people with dementia [60,61]. Another study found a significant improvement in word generation, an aspect of executive functioning, among people with dementia, as well as a trend toward improvement in most other cognitive measures. However, this trend did not reach statistical significance [62].

Finally, 27% (3/11) of the studies examined the potential of so-called exergames to improve cognitive and motor abilities in people with MCI [98,99] and dementia [111]. They developed a 12-week cognitive-motor training program incorporating different technologies—such as pressure-sensitive mats, videos, and heart rate variability monitoring—to support physical activity games. It proved to be feasible and effective in comparison to treatment as usual.

#### Social Interaction and Activation Using Robotics

A systematic review and meta-analysis of nonpharmacological, technology-based interventions to treat the symptoms of dementia, which was also included in the Digital Media in Reminiscence Therapy section, found that most interventions designed to improve social interactions incorporated social robots, ranging from a humanoid robot that looked like a small boy to robotic pets [104]. The meta-analysis reported an overall large effect of technology-based interventions on depressive symptoms. A scoping review also suggested that the use of social robots such as robotic animals can lead to an improvement in quality of life and mood and reduce loneliness [63]. However, results were dependent on the type of social robot, the severity of dementia, and the living context of patients (eg, the most common social robot, the baby seal Paro, appeared to be more beneficial for people with advanced dementia). Ethical considerations of using social robots were discussed, such as whether their use infantilizes patients or dehumanizes dementia care [63]. This was one of the very few instances in which an ethical discussion was part of a study on the digital treatment of older adults with mental illness.

#### Miscellaneous Technology

A total of 3% (2/64) of the studies looked at how wearable technology can be used in the treatment of psychiatric disorders [88,89]. An intervention consisting of motivational interviews and personalized messages combined with the wearing of a device to track activity and heart rate led to an improvement in insomnia symptoms [88]. A vibrating belt that had been shown to successfully prompt healthy older adults to walk in a particular direction was tested in a sample of people with dementia and was found to be usable among participants with mild dementia, although their performance was not error free, with some participants missing signals to take a turn [89].

Another intervention looked at the effect of heart rate variability biofeedback on depression and anxiety symptoms. Biofeedback is the use of technology to enable control of physical functions such as heart rate that are usually automatic. Combined with breathing and relaxation exercises, heart rate variability biofeedback was shown to be associated with significant decreases in depression and anxiety symptoms [90].

Finally, a single study looked at the potential of VR as a diagnostic tool for cognitive disorders, finding that VR can be used to differentiate between healthy older adults and people with MCI [91]. In the study, participants were evaluated based on their performance in a virtual supermarket where they had to find and pay for specific items on a shopping list using a tablet.

### State of Research: How Effective Are Digitalized Treatment Options for Specific Mental Illnesses in Older Adults?

#### Dementia and MCI

Dementia (37/64, 58%) and MCI (5/64, 8%) together were by far the most frequently studied diagnoses in research on digital interventions for older adults. The most widely used measure to assess the severity of cognitive deficits was the Mini-Mental State Examination [112]. The interventions in this area can be broadly grouped into four categories: interventions to (1) reduce psychological and behavioral symptoms and improve well-being, (2) measure and improve cognition, (3) support completion of activities of daily living, and (4) promote social interaction.

According to a meta-analysis of 16 trials, technology-based interventions for people with dementia were effective in reducing psychological and behavioral symptoms of dementia such as depression (large effect) and agitation (moderate effect), whereas no positive effects on apathy, anxiety, or neuropsychiatric symptoms were observed [104]. We found many applications of multimedia technology to reminiscence therapy for dementia, but a systematic evaluation of their effectiveness was missing [63,105,108]. Many interventions aiming to improve well-being among patients with dementia (8/42, 19%) applied personalized content—for example, personalized videos or music playlists—to evoke positive feelings and bring about changes in behavior among participants [53,55-57,71,81,83,100]. While these interventions were described as feasible and enjoyable [56,57,71,83,100] and there was some evidence of a significant reduction in symptoms of depression [56] and agitation [71,81], other improvements were either not significant or not systematically evaluated [53,56,71,83,100].

Interventions designed to promote cognition either were computer based [70,86,87,92,102] or used VR [60-62] and exergames [98,99,111]. Multiple studies (7/42, 17%) uncovered significant improvements in cognitive domains such as decision-making [62], immediate and delayed recall [70,99], general cognition [87,99,111], and attention and visuoconstruction [87] following digital interventions. However, results on other measures were inconsistent, and other studies (5/42, 12%) did not identify any statistically significant cognitive changes [60,61,86,92,102]. A study examining the feasibility of an online cognitive stimulation group reported limited competencies and access to technology as potential barriers to effectiveness [92]. One study examined the potential of VR for cognitive assessments and was able to correctly classify 91.8% of patients with MCI based on their performance in a virtual supermarket environment [91].

Several studies (8/42, 19%) demonstrated that audio or video prompts [47-52,77,78] as well as wearables [89] can be used to assist people with dementia in completing activities of daily living and finding their way. While participants accepted the technology and showed improvements in correctly performing activities of daily living while using the technology, the quality of the studies was low, with no control condition in any of the studies and small sample sizes (N=3-12).

Interventions aiming to promote social interaction using technology were varied, using multimedia systems to promote communication with caregivers or partners [57,84,100], social robots [63,104], and freely available apps within group therapy [66,80]. Overall, the investigated technologies were deemed feasible, but there was a lack of systematic evaluation studies [63,66,80,100]. There was some inconclusive evidence that multimedia systems may be associated with improved communication between people with dementia and their caregivers [63] and that they can act as a basis for joint attention during a shared activity [84]. The use of a social robot in dementia care showed high potential for improving social interaction and communication, but their beneficial effect was dependent on context, and their use poses ethical questions such as the potential dehumanization of dementia care [63,104].

#### Anxiety

Of the 10 interventions to treat symptoms of anxiety in older adults, 6 (60%) were iCBT interventions [45,46,72,74,76,101], and 1 (10%) was a relaxation technique delivered via video [58,59,94,95]. While 40% (4/10) of interventions were designed to treat both depression and anxiety [45,46,74,76], 33% (2/6) of the iCBT interventions were specifically tailored to patients with generalized anxiety disorder [72], with one of them designed for patients with comorbid pulmonary fibrosis [101]. A wide range of measures were used to assess anxiety, including the Geriatric Anxiety Scale [113], the State-Trait Anxiety Inventory [114], and the Geriatric Anxiety Inventory [115]. Apart from one study that examined the feasibility of the video-delivered relaxation technique [59], all studies (9/10, 91%) were evaluation trials of an overall high quality with control conditions in most cases [45,46,58,72,74,76,101]. The systematic evaluations revealed that digital interventions to treat anxiety were moderately to highly effective in reducing anxiety symptoms [45,46,58,72,76,101] as well as symptoms of depression [45,46,58,72,74], psychological distress, and disability [45,76]. The limitations discussed for iCBT for depression regarding limited follow-up measurements and the age of the samples also applied to iCBT for anxiety disorders.

#### Depression

All the original studies investigating interventions to treat depression (9/9, 100%) used an iCBT format. The quality of the studies was relatively high, with >50% of the studies (5/9, 56%) being randomized or nonrandomized controlled trials [46,73-75]. The severity of depressive symptoms was assessed using the Patient Health Questionnaire–9 [116] and the Geriatric Depression Scale [117]. Completion rates for iCBT courses ranged between 70% and 90%, indicating a high feasibility. iCBT for the treatment of depression in older adults was associated with a significant reduction in depressive symptoms with moderate to large effect sizes [44-46,69,73-75], as well as with a reduction in psychological distress and disability [45,75,76].

A limitation of the existing research on iCBT is that several studies (3/9, 33%) only measured changes up to 3 months after the intervention [44,45,73], making it difficult to ascertain whether improvements were maintained in the longer term, although one study found that improvements were maintained at the 12-month follow-up [46]. In addition, there was little research on the effectiveness of these interventions among individuals of very old age, with most research (6/9, 67%) focusing on participants in their 60s [44-46,69,73,75]. Furthermore, although one study ascertained that iCBT training from nonspecialist coaches can effectively treat depression [69], it is as yet unclear whether findings related to the general population that guided interventions are more effective than self-guided interventions [118] but as effective as face-to-face therapy [119] also apply to older adults. With the exception of one study [93], all others (8/9, 89%) focused on iCBT delivered via web applications accessed on computers; only 11% (1/9) of the studies examined a more modern mobile format, namely, a CBT smartphone app [93].

#### Sleep Disorders

A meta-analysis summarizing findings from 6 RCTs and 4 quasi-experimental studies reported that iCBT interventions were an effective way of treating insomnia [106] and led to an improvement in symptoms of insomnia (as measured using the Insomnia Severity Index [120]). Furthermore, 3 RCTs demonstrated longer sleep time in those that underwent iCBT training [106]. In another self-guided iCBT intervention for insomnia, it was found that improvements in objective and subjective sleep outcomes only applied to older adults with sleep complaints—poor sleepers who did not complain about their sleep did not reap the benefits of the training [67]. Another study investigated the effect of an intervention designed to increase physical activity that consisted of motivational interviews and wearing a device to track activity and heartbeat. The intervention was associated with a significant improvement in Insomnia Severity Index scores [88]. The limitations discussed for iCBT for depression regarding limited follow-up measurements and the age of the samples also applied to iCBT for sleep disorders.

#### Other Mental Illnesses

Another 3% (2/64) of the studies looked into technology-based interventions to treat older adults with mental illness in general [85,90]. Both studies were uncontrolled trials, so their findings need to be interpreted with caution. An existing self-management intervention for older adults was adapted for delivery via smartphone and tested among participants with various mental disorders [85]. The intervention was found to be usable in this target group, but the study did not systematically evaluate the effectiveness of the intervention. The other study evaluated the effectiveness of heart rate variability technology in biofeedback sessions and found significant pretest-posttest reductions in symptoms of depression and anxiety when combined with relaxation techniques [90].

## Discussion

### Principal Findings

The aims of this scoping review were to (1) identify which digitalized treatment options are available for older adults with mental illness, (2) chart the state of research on the feasibility and effectiveness of these treatments, and (3) derive potential directions for future research on the topic. On the basis of our search strategy and inclusion criteria, we found a total of 57 original studies and 7 reviews.

Regarding aim 1, we grouped the identified digitalized treatment options for older adults with mental illness into 9 different categories (Table 1). Web-based iCBT programs were the most common and designed to treat depression, anxiety, and sleep disorders in older adults. iCBT is attractive for the treatment of older adults as it can be conducted remotely, in their own homes, and at their pace. Moreover, online interventions can be adapted to the lived realities of older adults (eg, by integrating examples from their everyday life into the texts and assignments), making them more relatable. It should be noted that some older adults with mental illness may benefit more from in-person CBT sessions considering that loneliness and social isolation are a major burden in this age group [23,121]. One study found that, despite years of tailoring a digital intervention for older veterans, its effectiveness was limited by low motivation to engage in self-directed home practice, indicating that increased accessibility through technology did not necessarily address the underlying barriers to engagement [95]. Nevertheless, for individuals with limited mobility or no access to a human therapist, iCBT remains an appealing alternative. Acknowledging these differing needs, one study suggested that triaging could help identify those most likely to benefit from iCBT while directing others to offline interventions better suited to their circumstances [97].

Most of the other identified interventions for older adults with mental illness relied on basic technology such as audio, video, or digital photos to address the specific needs of people living with dementia, namely, inactivity, disorientation, emotional distress, and loneliness. Audio recordings were used to help patients successfully conduct domestic work in the home but also to simulate the calming presence of a family member in stressful situations. Existing treatment programs such as reminiscence therapy or other behavioral activation interventions for people with dementia were complemented with digital media, allowing for more engaging, personalized materials such as photos, videos, and music. An important finding of this review is that very few studies (7/64, 11%) dealt with more advanced technology such as AI, VR, or robotics. This is surprising as VR applications are increasingly being investigated in mental health [6] and appear to be feasible in the treatment of older adults with mental illness [122]. VR interventions are effective in treating posttraumatic stress disorder, anxiety disorders, and substance use disorders—all of which are prevalent in older adults [123]. However, all VR studies in our review (4/64, 6%) focused on dementia. In addition, despite the potential of AI technology in mental health, especially for serious mental illness [124,125], only 2% (1/64) of the included studies applied AI technology [79]. AI can be used for multiple aims in mental health, such as early detection and symptom monitoring, development of personalized treatment plans, analysis of medical health records, or social companionship. It should be leveraged to improve or create new treatment ideas tailored to the needs of older adults. Future studies exploring the usability of AI for older adults with mental illness could develop solutions for unsolved problems such as polypharmacy, loneliness, or loss of autonomy in this age group.

Concerning aim 2, the feasibility and effectiveness of the identified digitalized treatments, our review found some promising results as well as crucial gaps in the literature. The state of research on iCBT interventions for older adults reveals that these interventions are feasible, with satisfactory completion rates [76]. These interventions were systematically evaluated in controlled trials and shown to be moderately to highly effective in treating older adults with signs of depression, anxiety, and insomnia [44-46,67,69,72-76,88,106,109]. However, we identified several limitations of the state of research on iCBT for older adults with mental illness, namely, short follow-up periods in the evaluation trials, a lack of research with older patients (especially those aged >80 years), and a gap in the literature concerning comparisons of iCBT with and without guidance and its effectiveness compared to face-to-face treatment. Therefore, further research is needed to assess whether online interventions remain effective over time and are as effective in treating individuals of very old age and to what extent the level of guidance affects their outcome.

Another finding of our review was that the feasibility of integrating basic digital technology into dementia care is well established. Concerning the effectiveness of digitalized treatments, some studies (21/42, 50%) found that they alleviated symptoms of depression and agitation [56,71,81] and improved cognition [62,70,87] and completion of activities of daily living [47-52,77,78,89] as well as social interactions [57,63,66,80,84,104]. However, it should be noted that most of this research lacked systematic evaluation, with an abundance of original studies (25/37, 68%) having small samples or no control condition. As the feasibility of digitalized treatments in dementia care has been proven, future studies should take this research to the next level of systematic evaluation, investigating the effectiveness of these treatments with larger samples in controlled settings.

Furthermore, older adults with mental illness face specific barriers which limit the effectiveness of digital interventions. Limited digital skills and access to technology were reported as key challenges in samples from Brazil and India [92], and difficulties handling technical issues were reported as challenges in a study from Hong Kong [96]. These barriers are likely more broadly relevant given the negative correlation of digital competence with age and mental illness [14,111,126].

Finally (aim 3), we will summarize the identified gaps in the literature as well as directions for future research, which are also presented in Table 3. The summary of findings of the included studies reveals, first, a lack of research on how advanced technologies can be leveraged for the treatment of older adults with mental illness, especially VR and AI technologies. Future research should explore how these technologies can be used to address specific needs of older adults and what, if any, adaptations are needed to ensure the feasibility and usability of digitalized treatments among older adults. Second, the current state of the art neglects many mental disorders that are prevalent among older adults, prioritizing research on dementia care. While cognitive disorders are a relevant topic in older age, other mental disorders such as affective and anxiety disorders, substance abuse, psychosis, and other serious mental illnesses are highly prevalent in old age [123]. As digital interventions have been shown to be feasible and effective in treating serious mental illness in younger populations [1,6,11], older individuals with these conditions also deserve to benefit from the newest technological advances in treatments. Third, it was outlined that many of the included studies (32/57, 56%) were conducted with small sample sizes and without control groups. Moreover, the distribution of countries appeared imbalanced, with an overrepresentation of studies from North America and Italy and only 2% (1/64) of the studies being from Asia and none being from Africa. While one can only speculate about the reasons for this (eg, different distribution of age groups and resources between countries), the field would benefit from more diverse research samples as the findings of this review may not be generalizable. Thus, future research on the feasibility and effectiveness of all digitalized treatment options for older adults needs to be more systematic, with larger, more diverse samples and controlled study designs. Fourth, while our review found several examples of how technology can be tailored to meet the specific needs of older adults with mental illness, there is still a huge potential for personalized interventions in this field in dementia care and beyond. Future studies should explore how technology may solve the issue of many older adults with mental illness being isolated [21,22], experiencing more obstacles in finding professional mental health care [30,31], and having limited access to technology and digitalized treatments [10,13-16]. By developing strategies to bridge the digital divide, digital mental health care should be made accessible to all. Fifth, ethical considerations were insufficiently addressed in the included studies. As digital technologies become increasingly integrated into mental health care, it is essential to critically examine ethical concerns, particularly in vulnerable populations such as older adults with mental illness. Key issues include data privacy and security, transparency, algorithmic bias, informed consent, and appropriate use. Considering the biomedical ethical principles articulated by Beauchamp and Childress [127]—autonomy, beneficence, nonmaleficence, and justice—overreliance on technology or its improper use may cause more harm than benefit when they inadvertently undermine patient autonomy or exacerbate inequalities. If there is a significant gap in technology knowledge, this can also reinforce a power imbalance between younger clinical professionals and older patients. Importantly, technology should never infantilize older adults or replace meaningful human interaction, as outlined by one study on robotics [63]. Therefore, the ethical implications of digital interventions must be evaluated on a case-by-case basis. Technologies that may be unproblematic or even empowering for younger, technology-literate users could pose significant risks to older individuals unfamiliar with such tools. Future studies, especially on more advanced technologies such as AI, need to consider the ethical underpinnings of their research to ensure that older adults with mental illness can safely benefit from digitalized treatments. One promising approach is the inclusion of human-in-the-loop oversight, a key component of trustworthy AI [128]. Such oversight ensures that human judgment remains part of the decision-making process, reducing the risks associated with automated systems. The identified gaps in the literature show that the “digital gray divide” exists in mental health care and needs to be addressed to foster more equity between the age groups.

The results of this scoping review must be interpreted in light of some limitations and do not claim to be complete. Due to the exclusion of gray literature, conference proceedings, or other sources in this review, important findings may have been missed. The search strategy for this review was deliberately kept broad to provide an overview of as many different digitalized treatments as possible. Nevertheless, future reviews may use more specific search terms to include specific technologies such as AI. Another limitation of this review was that we only searched the PubMed database—including other specialized databases (eg, PsycINFO) may have yielded more articles and should be considered in future, more comprehensive reviews. Future reviews should also consider expanding the search strategy to include search terms that were not covered by MeSH terms. In addition, the references of the systematic reviews found in the initial search were not searched on the assumption that the most important findings would already have been summarized in the reviews themselves, which may have led to the exclusion of some relevant studies. Moreover, while excluding a formal risk-of-bias assessment is in alignment with the PRISMA guidelines for scoping reviews, it would have provided added value to the interpretation of the findings. Finally, the process of study selection could have been further improved by including a third author to resolve potential disagreements.

**Table 3:** Identified gaps in the literature and directions for future research.

Gap in the literature	Directions for future research
Lack of research on more advanced technology	Explore the potential of VR^a^, AI^b^, and other advanced technologies to address the specific needs of older adults. Investigate necessary adaptations for feasibility.
Lack of research on prevalent mental disorders in older adults	Expand research beyond dementia to include affective disorders, anxiety disorders, substance use disorders, psychotic disorders, and other serious mental illnesses. Replicate findings of digitalized treatments for younger populations.
Lack of systematic research	Conduct (randomized) controlled evaluation studies with large and diverse samples to assess the feasibility and effectiveness of digitalized treatments among older adults with mental illness.
Accessibility and equity	Develop personalized solutions to the specific needs of older adults with mental illness, especially strategies to bridge the “digital gray divide” and ensure equitable access.
Ethical considerations	Address ethical concerns such as data privacy, data security, transparency, bias, autonomy, and human connection in the development and implementation of digitalized interventions for older adults.

^a^VR: virtual reality.

^b^AI: artificial intelligence.

### Conclusions

In conclusion, this scoping review has demonstrated that there is a wide range of applications of digital technology available to treat mental illness in older adults, generally proving it to be feasible and accepted by patients. Results on the effectiveness of digitalized treatments vary and often lack systematic conformation. In addition, the state of research focused on few specific topics in the field, such as iCBT and basic technology in dementia care, neglecting other highly relevant problems for older adults with mental illness. We identified several critical gaps in the research on digitalized treatments for older adults with mental illness, highlighting a selective focus in research that exacerbates the “digital gray divide.” More equitable research is needed to ensure that all older adults with mental illness can benefit from the potential of digital interventions—addressing accessibility first, followed by expanding the research topics and methods. By leveraging advanced technologies in an ethical fashion, crucial challenges in the lives of older adults could be addressed and solved, improving their quality of life.

## Appendix

Multimedia Appendix 1 PRISMA-ScR (Preferred Reporting Items for Systematic Reviews and Meta-Analyses extension for Scoping Reviews) checklist.

Multimedia Appendix 2 Original studies included in the scoping review.

Multimedia Appendix 3 Review articles included in the scoping review.

## Abbreviations

AI: artificial intelligence
CBT: cognitive behavioral therapy
iCBT: internet-delivered cognitive behavioral therapy
MCI: mild cognitive impairment
MeSH: Medical Subject Heading
PRISMA: Preferred Reporting Items for Systematic Reviews and Meta-Analyses
RCT: randomized controlled trial
VR: virtual reality
